# Appealing to Faculty Gatekeepers: Motivational Processes for Intentions to Adopt an Evidence-B ased Intervention

**DOI:** 10.1093/biosci/biac029

**Published:** 2022-06-01

**Authors:** Peter McPartlan, Dustin B Thoman, Jennifer Poe, Felisha A Herrera, Jessi L Smith

**Affiliations:** San Diego State University, San Diego, California, United States; San Diego State University, San Diego, California, United States; University of Colorado, Colorado Springs, Colorado, United States; San Diego State University, San Diego, California, United States; University of Colorado, Colorado Springs, Colorado, United States

**Keywords:** faculty decision-making, faculty motivation, expectancy value cost theory, evidence-based classroom interventions, broadening participation

## Abstract

What motivates faculty teaching gateway courses to consider adopting an evidence-based classroom intervention? In this nationally representative study of biology faculty members in the United States (N = 422), we used expectancy–value–cost theory to understand three convergent motivational processes the faculty members’ underlying intentions to adopt an exemplar evidence-based classroom intervention: the utility value intervention (UVI). Although the faculty members perceived the intervention as valuable, self-reported intentions to implement it were degraded by concerns about costs and lower expectancies for successful implementation. Structural equation modeling revealed that the faculty members reporting lower intentions to adopt it tended to be White and to identify as male and had many years of teaching or were from a more research-focused university. These personal, departmental, and institutional factors mapped onto value, expectancies, and cost perceptions uniquely, showing that each process was a necessary but insufficient way to inspire intentions to adopt the UVI. Our findings suggest multifaceted, context-responsive appeals to support faculty member motivation to scale up adoption of evidence-based classroom interventions.

The United States relies on a steady supply of  highly trained bioscience researchers to develop new knowledge and translate discoveries to address public health concerns (National Institutes of Health 2012). Growing the scientific workforce is a top priority among funding agencies and professional societies (e.g., National Academies of Sciences, Engineering, and Medicine 2016), as is broadening participation among historically minoritized groups. Such growth necessarily depends on the pool of trained undergraduate students. Along the training pathway, foundational undergraduate courses in biology serve as a critical gateway to entering the science workforce. Indeed, attrition rates are significant within foundational science courses for all students (Mervis 2010), and they are substantially greater for minoritized students (African American, Latinx, Native American) in STEM fields (Chen and Soldner 2013, Garrison 2013, National Science Foundation 2019). Subsequently, scientists from these same racial and ethnic groups (as well as those who identify as Pacific Islander), are among those particularly underrepresented in biomedical fields (Valantine and Collins 2015). Performing poorly, losing interest, or otherwise feeling out of place in foundational coursework can lead students, especially underrepresented students, to switch majors, leave the sciences, or exit college altogether (Seymour and Hunter 2019, Rozensweig et al. 2021). This reality has led to calls to reimagine some aspects of traditional introductory bioscience education (e.g., the National Science Foundation's *VisionandChange* in undergraduate biology education program) that shift the focus from how to change students so they adapt to the learning context to how to change the learning context (Fox et al. 2009) in ways that are more inclusive and engaging to a diverse spectrum of students.

Social scientists and educational scholars have developed, studied, and disseminated various low-cost, empirically supported classroom interventions. These “wise” interventions show promising, sustained effects closing both opportunity gaps via strategies that support minoritized students’ motivation and engagement (Walton 2014, Harackiewicz et al. 2016, Casad et al. 2018, Walton and Wilson 2018), but there remains a disconnect between the evidence-based literature and the actual practice of adopting new teaching techniques (Handelsman et al. 2004, Henderson et al. 2011). So how do we, in the words of Brownell and Tanner (2012), convince “many faculty—not just a handful of faculty scattered across the country but the majority of life sciences faculty in every institution—to change the way they teach” (p. 339)? Our aim is to build on the existing frameworks for catalyzing change in biology education (Brownell and Tanner 2012, Owens et al. 2018, Herrera et al. 2020, Reihholz et al. 2021) by linking the motivational processes that biology faculty members experience to the personal, departmental, and institutional factors that can shape them.

Pedagogical transformation is more likely when faculty members see the need to change their classroom, know how to change it, and feel supported and incentivized for enacting change (e.g., Brownell and Tanner 2012, Owens et al. 2018, Bathgate et al. 2019). This notion fits well with an expectancy–value–cost conceptualization of motivation. Expectancy–value–cost theory (EVC) is especially useful for its ability to streamline how we understand the many distinct processes (expectancies, values, and cost) that converge to determine motivation for any given task. EVC predicts that a person's expectancies for success (i.e., “Can I do it?”), the perceived values of a task (i.e., “Do I want to do it?”), and its costs (i.e., “Is this worth it?”) are all necessary for understanding their motivation and behavior. The bulk of research on the theory has been focused on success expectancies (i.e., competence and confidence), although later refinements of the theory have better articulated the complexity of a task's value, articulating the types of value (Eccles 2013, Trautwein et al. 2013) and different cost concerns that factor into motivation (Flake et al. 2015).

For decades, scholars have used EVC to predict students’ educational choices and outcomes (e.g., Eccles 1987, Sullins et al. 1995, Eccles and Wang 2016). More recently, EVC has also been employed as a framework to study the motivation for engaging with new pedagogical practices among students (Cooper et al. 2017) and faculty members (Steinert et al. 2010, Matusovich et al. 2014, Bathgate et al. 2019), as well as to understand organizational change (Reihholz et al. 2021). Among faculty members, low expectancies of successfully implementing a new teaching practice appears to be the greatest impediment of motivation to adopt it (Bathgate et al. 2019, Orona et al. 2022). Faculty members’ confidence may be undermined without supportive colleagues and access to curricular resources (Matusovich et al. 2014, Bathgate et al. 2019). Faculty members are also more motivated when they see the value of a new teaching practice (Matusovich et al. 2014) and when the practice empowers their own professional goals, such as being rewarded during annual evaluations or promotion reviews for undertaking pedagogical innovations (e.g., Matusovich et al. 2014, Orona et al. 2022). Faculty members may also see more value in classroom practices that reaffirm their own identity as someone who engages in professional growth (Steinert et al. 2010). As would be predicted by EVC, adopting a new classroom practice also depends on faculty members’ perceptions of its costs. They recognize that, when it is not easy to implement a given practice, the time required to improve their teaching may be better spent on other activities, such as research, which can support their professional goals (Matusovich et al. 2014).

As we look to scale up interventions, can faculty members’ personal, departmental, and institutional characteristics help us understand their expectancies, value, and cost perceptions? EVC posits that personal, situational, and cultural forces may shape who is likely to perceive greater expectancies, values, and costs and the resulting behavioral motivation (Eccles and Wigfield 2002). At the student level, for example, we know that parents’ cultural beliefs that math will be harder for their daughters than for their sons have been linked to girls having lower expectancies for success in math and higher math anxiety (cost), ultimately reducing their motivation to take math courses (Eccles and Jacobs 1986). Although empirical work exploring these links among faculty members’ adoption of new pedagogies is in the early stages, extant research in other fields suggests several characteristics worth exploring. Social gender roles and cultural backgrounds likely influence the adoption of such strategies. For example, women and individuals from racially minoritized groups tend to be more motivated by communal values of helping and working with others than are White men, and this greater communal value orientation predicts greater interest in activities that help other people (Brown et al. 2015, Diekman et al. 2015, Thoman et al. 2015, Boucher et al. 2017), including mentoring and service activities that support underrepresented students (Williams and Dempsey 2014, Matthew 2016, Guarino and Borden 2017, Miller and Roksa 2019). It is also the case that contextual characteristics at departmental and institutional levels likely also contribute to faculty members’ knowledge, support, and incentive for enacting classroom changes (Kezar and Eckel 2002, Brownell and Tanner 2012). By showing a correlation of these characteristics with expectancies, value, and cost, EVC may reveal the nuances of how faculty members’ personal and contextual characteristics affect their intentions to adopt an evidence–based intervention.

## Present study and hypotheses

One difficulty in asking faculty members about their motivation to make pedagogical changes is knowing what they are considering when asked if they will change their classroom: Are they thinking about active learning (Allen and Tanner 2005), flipped classrooms (Sletten 2017), growth mindsets (Yeager et al. 2019), values affirmation (Miyake et al. 2010), or some other strategy? To address this concern, we focused our study on one exemplar, diversity–enhancing classroom intervention with a strong empirical base, the utility value intervention (UVI; Hulleman et al. 2010, 2017, Gaspard et al. 2015, Harackiewicz et al. 2016). In this way, we reduce the conflation between perceptions about pedagogical changes and the idiosyncrasies of different interventions. The UVI is a writing assignment that itself draws on EVC, asking students to write a 500–word essay summarizing the most recent class content while making explicit connections to how the information is useful in their own lives (the utility value of the information). The assignment is integrated into the course, occurs at regular intervals (typically, three times in a semester), and is worth a small amount of class credit.

The UVI was developed and tested in randomized double–blind trials in introductory biology classes at one university and has since been tested at universities and community colleges across the nation. Multiple studies show that the UVI increases biology student grades, improves motivation, increases the likelihood of the students enrollment in the next biology course, and predicts higher student retention in the science major over time (e.g., Canning and Harackiewicz 2015, Harackiewicz et al. 2016, Rosenzweig et al. 2018, Hecht et al. 2019). Moreover, the UVI is particularly appealing to study as an exemplar intervention because of its especially positive effects for minoritized and first–generation college students. The UVI reduced the opportunity gap by some 61% for these students compared with their White and Asian continuing–generation peers in foundational biology courses (Harackiewicz et al. 2016).

Using this exemplar classroom intervention and the EVC framework, we set out to test how faculty members’ perceived expectancies for the success, value, and cost of such an intervention were related to their motivation to implement it. Drawing from EVC, we hypothesized that biology instructors’ motivation and implementation intentions for the classroom intervention would be positively correlated with their expectancies for successfully implementing it and their perceptions of its value but negatively correlated with their concerns about its costs.

We further investigated how the faculty members’ personal, departmental, and institutional characteristics shaped their motivation. We predicted that the associated expectancies, values, and costs would mediate the relationship between the characteristics and implementation intentions for the intervention. Specifically, we expected personal characteristics, such as gender identity and identifying with a racially minoritized group, to predict the faculty members’ valuation of the intervention. We also predicted that departmental characteristics (such as class size and teaching workload allocation) and institutional characteristics (such as the university's research emphasis) would be significantly correlated with cost concerns (Matusovich et al. 2014, Bathgate et al. 2019). We tested our hypotheses by presenting the UVI to a random, nationally representative sample of US biology faculty members who had recently taught foundational biology classes and asking them to report their motivation for and their likelihood of implementing the intervention the next time they teach the course.

## Participants and procedure

This study was conducted among a nationally representative sample of instructors of biology courses in the United States. To identify our faculty participants, we created a database by random sampling a list of 800 4–year universities and colleges from among the more than 40,000 institutions in the Integrated Postsecondary Education Systems (IPEDS) database (see section A in the supplement). We used publicly available websites to determine the contact information of faculty members teaching introductory biology courses, inviting 3390 faculty members to participate. Our response rate was 16.4%, which was expected considering the cold contact method used among a faculty population. The represented gender and racial or ethnic identities of these faculty participants reflected those of earned doctorates in life sciences (National Science Foundation 2019). Because respondents and nonrespondents typically do not significantly differ when demographics of respondents match those of the underlying population, generalizable findings could be reasonably assumed (Holbrook et al. 2007).

After considering exclusion criteria (see section D in the supplement), our analysis sample was *n* = 422. Participants ranged in age from 28 to 77 (mean [*M*] = 47.7, standard deviation [SD] = 10.0) with a relatively even number identifying as male (47.2%) and female (50.0%) faculty members (*n* = 11 respondents did not indicate their gender, and 1 indicated a nonbinary gender identity). The sample was largely White (82.7%), with 9.4% identifying with minoritized backgrounds. *Minoritized* was defined as American Indian or Alaska Native (1.7%), Black or African American (2.1%), Hispanic or Latino (5.5%), or Pacific Islander or Native Hawaiian (0.0%), aligning with data suggesting that people with these racial or ethnic identities are particularly underrepresented in faculty positions related to the biological sciences (Valantine and Collins 2015). In addition, 6.2% of the faculty members were Asian or Asian American, and 1.2% were Middle Eastern. The participants were from 182 distinct universities. Of note, 68.7% of these universities were represented by just one or two faculty members (*M* = 2.3, SD = 1.86, median = 2, range = 1–12). Among the faculty members, 28.7% were from minority-serving institutions. Aligning with recent work identifying African-American, Latinx, and Native American students as minoritized in fields of biological science (Chen and Soldner 2013, National Science Foundation 2019), historically Black colleges and universities, Hispanic-serving institutions, and tribal colleges were accordingly considered *minority serving institutions*. Finally, 48.1% were at a doctorate-granting, Carnegie Research 1 university (R1, rated as having very high levels of research activity; see section A in the supplement).

As part of a larger study of pedagogical decision-making (see section E in the supplement), randomly selected biology faculty members from our participant pool were sent an introduction email inviting them to participate in a National Science Foundation– “funded study researching biology faculty perceptions about course materials and teaching practices” in exchange for a $30 gift card. All participants watched a brief video and read material describing the intervention, including a description of and references to articles supporting the evidence-based benefits to biology students’ grades, science interest, and persistence in science (see section B in the supplement).

After learning about the intervention, the participants completed survey measures in a counterbalanced order of personal characteristics (e.g., gender identity, racial or ethnic identity, teaching experience); departmental characteristics (e.g., size of classes, percentage of workload dedicated to teaching); and expectancy, value, and cost measures, as well as two key outcome scale variables: self-reported implementation intentions (e.g., “Estimate the likelihood that you will implement the UVI the next time you teach a course” on a percentage scale and “Could you see yourself leading this classroom intervention: yes, maybe, or no?”) and motivation for the intervention (e.g., “I would enjoy using this intervention in my classes”). Institutional characteristics, including the percentage of enrolled students from minoritized backgrounds (American Indian or Alaska Native, Black or African American, Hispanic or Latino) and the percentage of the fiscal year 2018 budget from research expenditures were gathered from the IPEDS database (see section C in the supplement).

## Predicting faculty motivation

Overall, the descriptive data (table 1) showed that the faculty members self-reported their belief that they were generally motivated to implement this intervention, with especially strong agreement about its value. Each of our dependent variables was measured on a 1–7 Likert scale for which 1 indicated strong disagreement, 7 indicated strong agreement, and 4 indicated neither agreement nor disagreement. One-sample *t*-tests showed that the average of each measure was significantly different from the scale midpoint of 4, confirming the faculty members’ generally positive opinions about the intervention. They agreed that they felt able to implement the intervention (*M* = 4.61, SD = 1.29), found it valuable (*M* = 6.07, SD = 0.65), experienced motivation for trying the intervention (*M* = 5.68, SD = 0.89), and intended to implement the intervention in their biology class (*M* = 5.35, SD = 1.44; df = 421, *p* < .001 for all tests). Indeed, 62.6% of the respondents indicated that they could see themselves leading this classroom intervention.

**Table 1.** Correlations among study variables and their descriptive statistics.

	Implementation intentions	Motivation	Expectancies	Value	Cost	Years teaching	Size of class	Percentage of workload dedicated to teaching	Percentage of minoritized student enrollment at the university	Percentage of budget from research expenditures
Implementation intentions	–									
Motivation	.62**	–								
Expectancies	.50**	.41**	–							
Value	.47**	.65**	.34**	–						
Cost	–.54**	–.45**	–.68**	–.41**	–					
Years teaching	–.13**	–.15**	–.11*	–.10*	.12*	–				
Size of class	–.16**	–.06	–.27**	.00	.13**	–.08	–			
Percentage of workload dedicated to teaching	.01	.05	.03	.10	.03	–.05	–.01	–		
Percentage of minoritized student enrollment at the university	.08	.06	.04	.09	–.08	–.10	.05	.02	–	
Percentage of budget from research expenditures	–.15**	–.12*	–.17**	–.11*	.07	.0047	.50**	–.32**	–.10*	–
Cronbach's alpha	.82	.90	.83	.69	.90					
Mean	5.35	5.68	4.61	6.07	3.55	15.35	1.94	62.46	27.89	5.47
SD	1.44	0.89	1.29	0.65	1.41	9.66	0.99	22.46	18.89	7.74
Response range	1–7	1–7	1–7	4–7	1–7	1–50	1–5	0–100	0–93	0–43

*Note: n* > 405 for all pairwise correlations (less than 5% missing data from total sample *N* = 422). The first five variables are multi-item scales measured from 1 to 7. The size of the class is coded as 1, *less than 50*; 2, = *50–149*; 3, *150–300*; 4, *300–499*; 5, *more than 500*. *p < .05. ** *p* < .01.

However, this did not mean that the intervention was believed to come without costs related to resources, time, and grading. The average response about cost perceptions was significantly below the scale midpoint, indicating slight disagreement with the idea that the intervention would take too much effort (*M* = 3.55, SD = 1.41; df = 421, *p* < .001). But the relatively large standard deviation shows that many of the respondents did agree that the UVI had substantial costs despite its value. To gain a more concrete understanding of these costs, we analyzed additional descriptive data from the 37.4% of the respondents who indicated reluctance to implement the intervention (i.e., who answered *no* or *maybe* to the question *“*Could you see yourself leading this classroom intervention?”). Of these respondents, 72.8% (27.3% of the entire sample) said that the most important reason for their reluctance was concern over a lack of resources, time, or grading.

**Figure 1.**
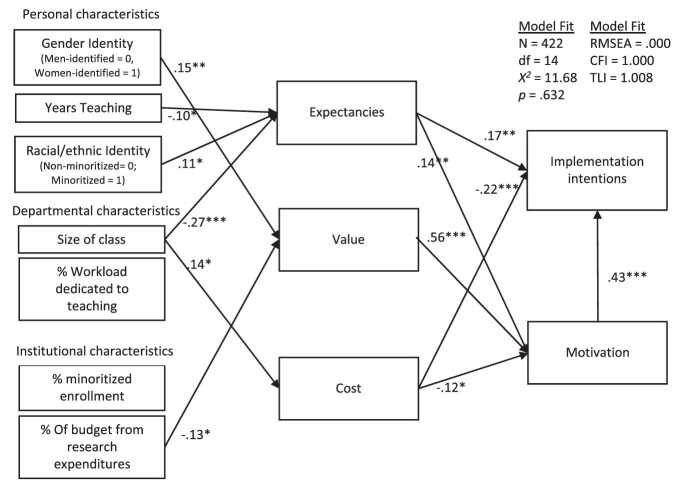
Structural equation model of the effects of various faculty characteristics on implementation intentions, as mediated by expectancies, values, and costs. Insignificant paths are not shown. Exogenous variables are all correlated. The residual variances of all mediators (expectancies, value, cost) are correlated.

We next used structural equation modeling to examine the roles that expectancies, value, and cost each played in determining the respondents’ likelihood of implementing this intervention. We used STATA 15 (StataCorp 2017) to model the effects of expectancies, value, and cost in predicting these outcomes. Simultaneously, expectancies, value, and cost were mediators of the relationship between the respondents’ characteristics and their implementation intentions. As we predicted, a good-fit model demonstrated that expectancies, value, and cost all mediated at least one relationship between the instructors’ characteristics and their implementation intentions (see figure 1). The indirect effect of value on implementation intentions through motivation was significant (*ß* = .24, 95% confidence interval [CI] = .18–.30), and this indirect effect alone was similar in size to the total effects of expectancies (*ß* = .23) and cost (*ß* = –.27).

For this intervention, the uniquely strong correlation between expectancies and cost (*r* = –.68; see table 1) led us to conclude that these beliefs drove implementation intentions more than value. Their strong correlation alludes to a likelihood that faculty members who are worried about time and grading for this writing intervention (cost) may not be confident they can implement it correctly (expectancies) and vice versa. Such a high correlation between these two processes presented the idea that, although our model separates these effects on a theoretical basis, it may be important to recognize them as a similar process on a practical basis. To test this idea, we ran two additional models: one that removed expectancies and one that removed cost (see section D in the supplement). As we anticipated, the total effect of expectancies on implementation intentions jumped to *ß* = .39 (95% CI = .31–.47; model S3), and the total effect of cost jumped to *ß* = –.42 (95% CI = –.49 to –.32; model S4). In either case, the impact of these expectancy or cost perceptions on implementation intentions was significantly stronger than that of value, which stayed the same (*ß* = .24). However, we still focus on the direct and indirect effects of the full expectancy–value–cost model in figure 1 because of the theoretical benefits of identifying different predictors of expectancies and cost and because model S3 or S4 did not fit the data significantly better.

As is illustrated in figure 1, the results reveal different motivational processes by which certain faculty members might be more likely to implement this intervention. First, the instructors of smaller classes had lower cost concerns and higher expectancies of success. The instructors with fewer years of teaching experience and those from minoritized backgrounds also had higher expectancies for successfully implementing the intervention. Female-identified faculty members and those at universities that spend less of their budget on research were also more likely to implement the intervention. The results show that this was because the respondents from these backgrounds perceived more value in the intervention. Overall, the faculty members who identified as women or who were racially minoritized, taught smaller classes, had fewer years of service, or were from a less research-centric university reported greater implementation intentions for the intervention. The further statistical analyses detailed in section D of the supplement show that the differences in implementation intentions were statistically significant with respect to gender identity, class size, years of service, and institutional research.

## Implications for scaling evidence-based classroom interventions

In this study, we sought to identify helpful information for instructors and intervention scientists who want to scale up the adoption of inclusive, evidence-based pedagogies. We leveraged a nationally representative sample of biology instructors of introductory courses at 182 universities across the United States and presented them all with the evidence-based example of such a pedagogy: the UVI. The results demonstrated generally high self-reported intentions to implement the exemplar intervention (the UVI), albeit dependent on three convergent motivational processes. As was predicted by EVC, the motivation and implementation intentions for the intervention were jointly influenced by the expectancies for success, the value of the intervention, and the cost concerns. Indeed, our data suggested that each factor was a necessary but insufficient way to inspire using this particular classroom intervention. For example, although there was universally strong agreement among the respondents that the UVI had value, that value alone was not sufficient to motivate their implementation intentions. The respondents’ cost concerns (e.g., resources and time) for the intervention went hand in hand with doubts that they would be able to easily implement it (expectancies), degrading their ultimate implementation intentions. Although determining the precise impact of these processes on actual implementation requires looking beyond self-reported implementation intentions and observing instructors’ actual use of the UVI over time, our data reaffirm the usefulness of EVC for understanding the key step of supporting instructors’ motivation for adopting evidence-based interventions to begin with.

Importantly, the results also demonstrated that the relative contribution of the three motivational processes differed as a function of the respondents’ personal, departmental, and institutional characteristics. In addition to taking these results at face value, it is worth connecting these findings to the decades of sociology and higher education research demonstrating how the importance of access to resources (defined broadly, funding, experience, and influence) shapes pedagogical experience, motivation, and outcomes (e.g., Torres and Mitchell 1998, Nichols and Stahl 2019). Using this lens, one interpretation of our data is that lower intentions to implement the UVI were self-reported by respondents with historically greater influence over shaping the field of biology (identified as male, White, with more years of experience, or from a university with more research funding). This interpretation points to the structural and historical forces that can shape motivation for change (e.g., Jost et al. 2004) and suggests that individual-level strategies alone will not lead to intervention adaption at great scale; structural and policy-based strategies will also be required.

Just as some groups of people are, on average, less receptive to research about bias in STEM (Handley et al. 2015), other groups of people are, on average, more likely to focus on topics related to minoritized group disparities (e.g., Shavers et al. 2005, Hoppe et al. 2019). Finding that some instructors’ personal and context characteristics are, on average, good predictors of their level of support for this one diversity-enhancing intervention is an important consideration for intervention and education scholars who are considering assumptions, the point of entry, or the approach one might want to use to make a convincing case about the need for pedagogical changes when working in different settings or with different groups of people (e.g., Flynn 2015, Smith et al. 2021). For example, prior studies indicate that women and faculty members of color spend more time preparing for and engaging in teaching than their White or male colleagues (Hurtado et al. 2012) and are more likely to engage students in active, student-centered pedagogies (Milem 2001, Umbach 2006, Eagan and Garvey 2015). Indeed, research shows that faculty members’ identities are often important in guiding the perceived value of a particular classroom intervention (Speed et al. 2019). Interventions that foreground cultural issues or racial opportunity gaps, such as difference education and sense-of-belonging interventions (Walton and Cohen 2011, Stephens et al. 2014), may resonate more with those faculty members who themselves identify with marginalized groups, although, of course, this is not always the case. Whereas this study, which was focused on decision-making about the UVI, specifically, implicated resource concerns (costs) as a major hurdle to implementation, the motivation to implement other interventions may hinge more on how the faculty members’ identities shaped the perceived value of the intervention.

Our findings further confirm that institutional contexts can influence pedagogical practices and decisions when considering interventions such as the UVI (Myers and Myers 2015). Our results illustrated that adopting the UVI in biology classrooms at large R1 universities, for example, would likely require preemptively assuaging time and effort cost concerns. Research from biology education underscores that collective action through department-wide strategies is important in this regard (Owens et al. 2018). Combing collective actions to create institutional cultures that value the two-way relationship between teaching and research (Reid and Gardner 2020) with individual-focused policies that incentivize pedagogical innovation with promotion, tenure, and annual review processes could lower costs. In addition, providing more templates and resources may improve expectancies for success, especially for instructors with large class sizes. Meanwhile, smaller, more affordable steps can be immediately taken by departments to raise the expectancies for success, such as actively recognizing and discussing new pedagogies that have been successfully employed (e.g., Owens et al. 2018). As barriers to pedagogical change at large research universities are being addressed, it is also useful to know where there may be greater motivation to readily adopt the intervention. Working with instructors at less research focused universities may create champions of the intervention who can provide more evidence of its effectiveness. More research focused specifically on discipline-based education researchers at large research universities may also reveal key roles that partnerships with those unique faculty members may play in scaling up intervention adoption. Understanding which features and contexts, on average, may predict more or less motivation to implement the intervention is useful to those wishing to persuade biology educators to make classroom changes at scale.

To be sure, instructors’ motivation to adopt a particular intervention depends on the intervention in question. In this study, we investigated a single exemplary intervention, the UVI, to ensure our analyses were not confounded by faculty members imagining different types of scenarios. We specifically chose the UVI because of its well-documented empirical support for biology students’ engagement, achievement, and persistence, especially among minoritized students. It was therefore encouraging to affirm that, among this nationally representative sample, the UVI was seen as highly valuable. A distinct characteristic of the UVI is its focus on writing. Assigning and grading short essays emerged as a major concern for our participants, despite the value of its effectiveness. Promoting writing within curricula, especially within the sciences, may require unique learner-centered and active-learning support structures (Ebert-May et al. 2011, Reynolds et al. 2012, D'Avanzo 2013). Using an intervention that is high in value, however, has limitations. We do not know how an intervention perceived to be low in value might undermine the instructors’ implementation intentions. For different psychological interventions that require less writing and time grading, expectancies and cost may be much less of an issue.

All told, the UVI is just one example of an evidence-based intervention worthy of adoption. As scholars attempt to scale up their interventions to additional classroom settings, EVC offers a framework for documenting promising pathways and identifying possible challenges to wide implementation. We also argue that different stakeholders must each take significant action to affect positive curriculum changes, no matter the type of intervention under consideration. However, we hope that concerns about the daunting nature of this task can be assuaged by leveraging precise motivational processes grounded in both theory and empirical evidence. For example, cementing the value of an intervention takes a commitment from funding agencies and educational researchers to appropriately test and confirm the intervention's effectiveness with empirical evidence. Likewise, university leadership can play a major role in shaping institutional structures. Leaders can reduce cost concerns by restructuring policies to reward pedagogical risk taking and innovation through promotion and tenure processes or annual review evaluation. Moreover, departments can work to create cultures that support expectancies for success by offering relief time, differential workloads, or additional teaching assistant support to faculty members who attempt to restructure courses to adopt new strategies. Ultimately, to meaningfully change student outcomes and grow the bioscience workforce, the community must be willing to support faculty members’ expectancies, value, and cost concerns, because instructors are the gatekeepers to their classrooms.

## Supplementary Material

biac029_Supplemental_File

## Author Biographical

Peter McPartlan (pmcpartl@uci.edu) is a postdoctoral research fellow and Dustin B. Thoman is an associate professor of psychology at the Center for Research in Mathematics and Science Education at San Diego State University, in San Diego, California, in the United States. Jennifer Poe is the program manager for this National Science Foundation–funded Adapt project; she is also an instructor of psychology and serves as the director of the Center for Student Research at the University of Colorado, in Colorado Springs, Colorado, in the United States. Felisha A. Herrera is the director of the Research and Equity Scholarship Institute and is an associate professor of postsecondary education at San Diego State University, in San Diego, California, in the United States. Jessi L. Smith is the associate vice chancellor for research and is a professor of psychology at the University of Colorado, in Colorado Springs, Colorado, in the United States.
