# Supplementary material for: Appealing to Faculty Gatekeepers: Motivational Processes for Intentions to Adopt an Evidence-B ased Intervention
Source: Bioscience. 2022 Jun 1;72(7):664–72. doi: 10.1093/biosci/biac029 (PMC9236873; doi:10.1093/biosci/biac029)
Supplement: biac029_Supplemental_File [file biac029_supplemental_file.docx]

**Supplemental Material**

**Appealing to Faculty Gatekeepers:**

**Expectancy, Value, and Cost Concerns for Adopting an Evidence-based Intervention**

Table of Contents

[Section A. Participants 2](#_Toc96610048)

[Section B. Procedure 5](#_Toc96610049)

[Section C. Measures 8](#_Toc96610050)

[Section D. Analyses and Robustness Checks 12](#_Toc96610051)

[Section E. Larger Study Details 18](#_Toc96610052)

[Section F. Additional Descriptive Statistics 20](#_Toc96610053)

# Section A. Participants


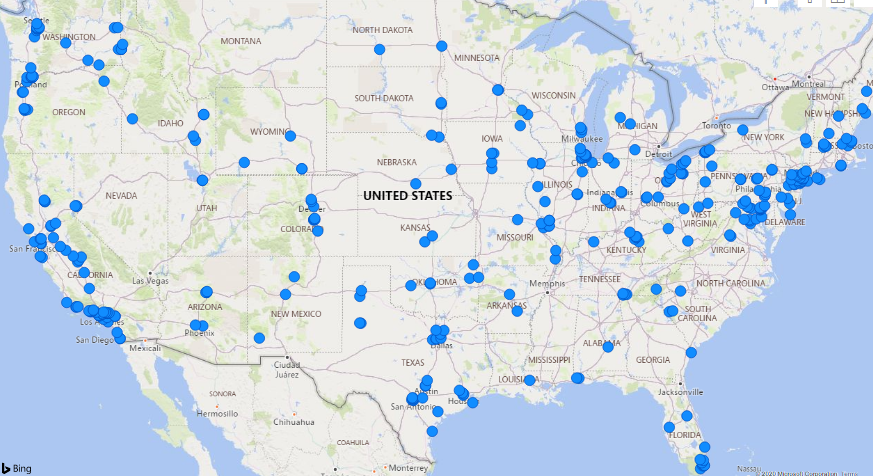

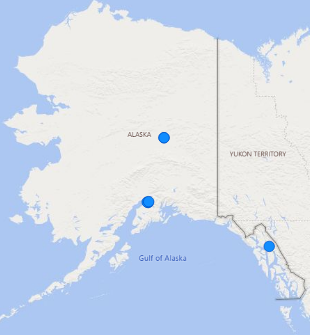
 We first created a large national database with contact information (i.e., names, emails) of several thousand potential biology faculty participants. Building this database occurred in a series of stages whereby a larger database of all institutions of higher education in the US was pared down to only those institutions offering a 4-year bachelor’s degree in Biology. This initial pool of institutions was taken from the Integrated Postsecondary Education Systems (IPEDS) database resulting in an initial list of over 40,000 schools. In order to make vetting these institutions more manageable, a randomized subset of 800 schools was taken. After confirming that the schools offered a 4-year degree in Biology, our team identified the faculty teaching introductory Biology courses, and entered the names and email addresses of these faculty to the database. This vetting process resulted in contacts for several thousand faculty members. Between September 2019 and July of 2020, we contacted 3,390 of those faculty members to participate in our study. “cold contact/email” Six hundred fifty-five faculty responded to the survey, creating a response rate of 16.4%.

**Figure S1.** Locations of institutions represented in participant database.

After data collection for the entire study was complete, we determined the 422 eligible, valid respondents came from 182 unique institutions from 49 US states (Figure S1). The percentage of these institutions that were from minority serving institutions and various Carnegie research classifications can be seen in Table S1. Notably, this table suggests that a relatively larger proportion of faculty participants came from doctoral universities. This is likely because doctoral universities have larger numbers of faculty in their biology programs, thereby drawing more faculty willing to participate in our study. In addition, Table S2 shows the racial/ethnic identities of faculty from minoritized groups were roughly equivalent to those of faculty across the United States (NSF 2017)

| **Table S1** | | | |
| --- | --- | --- | --- |
|  | | | |
| *Institutional Variables – Breakdown of Institutional Data by University and by individual faculty* | | | |
|  | | Faculty Represented in Sample | Unique Universities Represented in Sample |
| *Minority serving institution* | |  |  |
|  | Hispanic-Serving Institution | 28.20% | 31.49% |
|  | HBCU | 0.47% | 1.10% |
|  | Tribal College | 0.00% | 0.00% |
| *Carnegie classification* | |  |  |
|  | Doctoral | 48.10% | 41.01% |
|  | Master's | 38.63% | 43.82% |
|  | BA/Associate's | 11.61% | 15.17% |
|  |  |  |  |
| Observations | | 422 | 182 |

| **Table S2** | | | |
| --- | --- | --- | --- |
|  | | | |
| *Racial/ethnic identities of faculty in sample vs. all of U.S.* | | | |
|  | | Sample | All of U.S. |
| *Racially-minoritized identifying* | |  |  |
|  | American Indian/Alaska Native | 1.7% | 0.2% |
|  | Black/African American | 2.1% | 4.0% |
|  | Hispanic/Latino | 5.5% | 4.7% |
|  | *Pacific Islander/Native Hawaiian* | 0.0% | 0.1% |
| *Non-minoritized identifying* | |  |  |
|  | White | 82.7% | 69.7% |
|  | Asian/Asian-American | 6.2% | 20.0% |
|  | Middle Eastern | 1.2% | - |
| *Note*. Source is NSF report on science, engineering, and health doctorate holders employed full time as full, associate, or assistant professors in universities and 4-year colleges in 2019. | | | |

# Section B. Procedure

Before collecting data for each study, details of the procedure, measures, sample size, and analyses were pre-registered at AsPredicted.org (see links to PDFs in Section E). For each study, randomly selected biology faculty from our participant pool database were sent an introduction email with study details and forewarning them that they would be sent the study invite via email in the coming week. Participants were then invited to an “NSF-funded study researching Biology faculty perceptions about course materials and teaching practices” via a 20-25 minute survey on the Qualtrics survey platform in exchange for a $30 gift card. For all studies, participants first completed an online consent form and then were then asked to view a video and additional material describing the utility-value intervention (UVI).

All studies presented a description of the UVI, as presented through a narrated video with subtitles. The UVI was described as an evidence-based way to get engage students with classroom material by having them find their own connections between the course material and their own lives (e.g., goals, hobbies, future). After describing the gains associated with the UVI and students’ grades, interest, and pursuit of more science classes, more detail about the assignment was provided (e.g., the assignment should be given three times during the course, it asks students to generate their own question about a biological topic from class, and then write a 500-600 word essay answering that question while making connections with their lives). Following the video, participants viewed a text summary of the UVI’s benefits. These included higher course grades, increased interest in science, and greater likelihood of enrolling in future science courses.


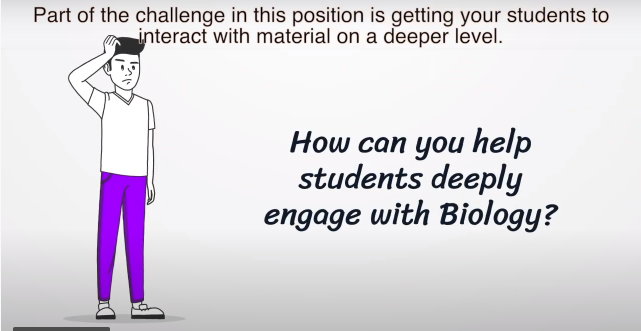

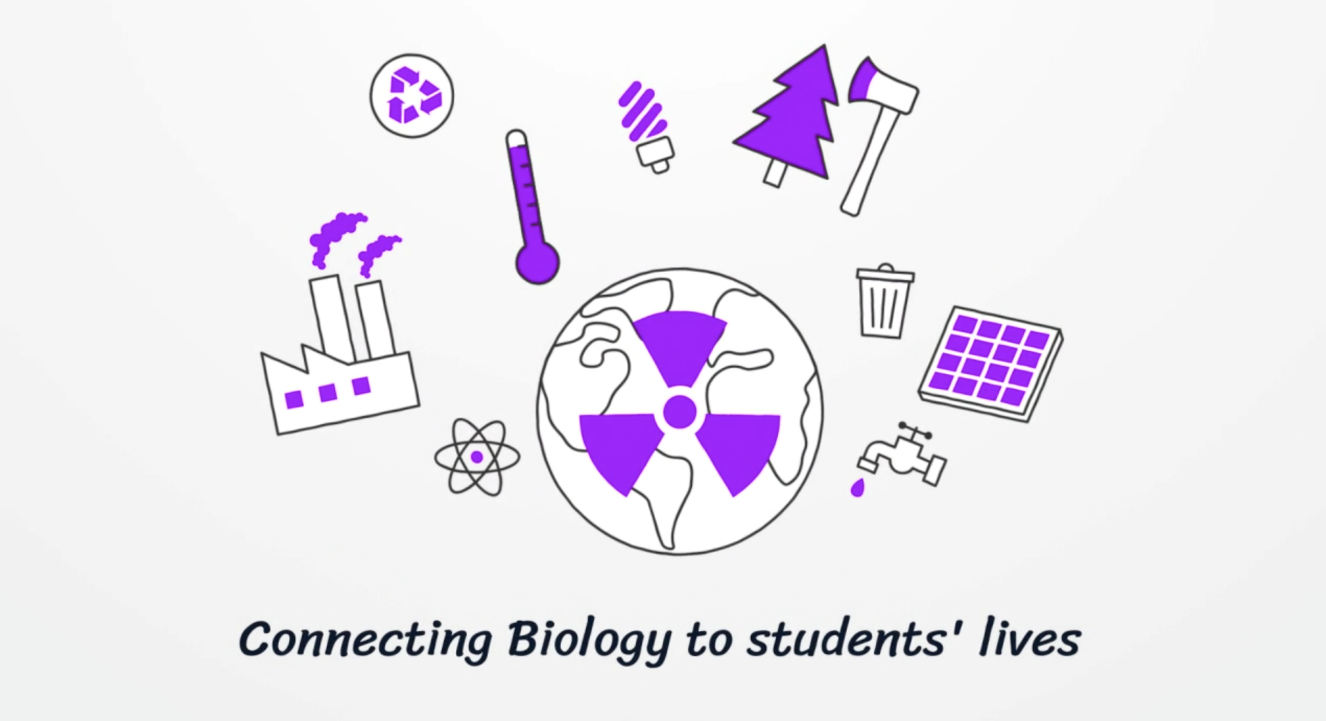


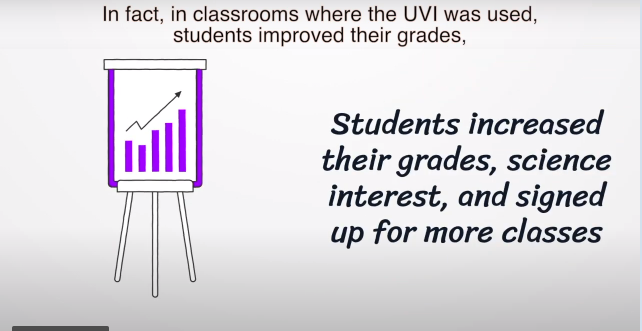

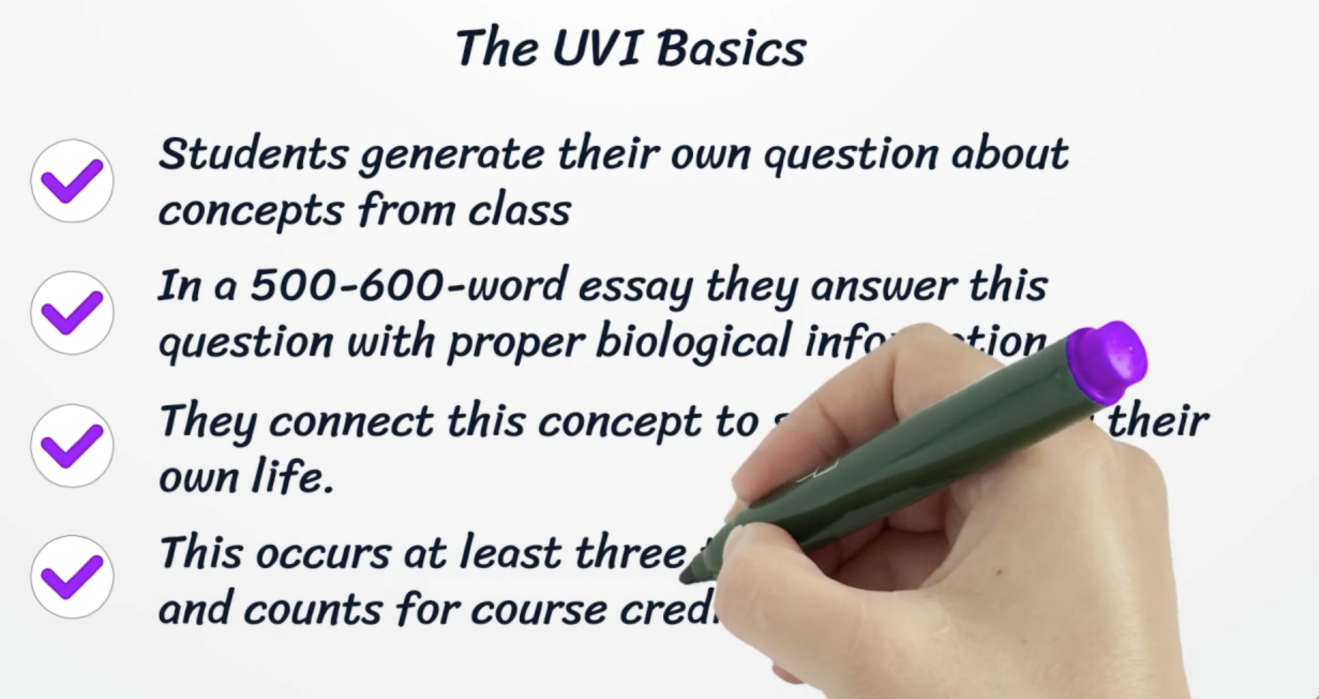


**Figure S2.** Screenshots of video describing the UVI.

Participants then viewed a static PDF version of the assignment in greater detail and completed open-ended manipulation and attention checks to ensure they reviewed the materials containing the manipulations.

Participants then completed a series of counterbalanced survey measures including the primary mediator and outcome variables (motivation variables), demographics and job characteristics (e.g., rank, years at the institution), as well as selected exploratory measures (e.g., growth mindset, system justifying beliefs). Following the survey, participants were debriefed as to the purpose of the study. They were also provided the option to view additional UVI materials and references supporting its use in science classrooms. After completing the survey, they were immediately linked to the website to claim a gift card incentive. The entire study session lasted approximately 25 minutes. Institutional characteristics were recorded separately using publicly available databases (i.e., IPEDS).

# Section C. Measures

**Primary Outcome Variables**

Our primary outcome variables were Biology faculty’s implementation intentions regarding the UVI and their intrinsic interest in the UVI. These were both multi-item scale measures composed of Likert-scale question, scaled to be from 1-7, with higher values indicating more of the respective construct. Because these measures were being adapted for a faculty sample for the first time, we conducted factor analyses after data collection but before treatment analyses. Details for each scale are below.

**Implementation intentions**. This was a three-item scale adapted from Carroll et al. (2009). The first two items, “Estimate the likelihood that you will implement the UVI the next time you teach a course,” and “Estimate the likelihood that you will learn more about the UVI in the next academic year” were measured on the following scale: 1 = *Extremely unlikely*, 2 = *Moderately unlikely*, 3 = *Slightly unlikely*, 4 = *Neither likely nor unlikely*, 5 = *Slightly likely*, 6 = *Moderately likely*, 7 = *Extremely likely*). The third item, “Could you see yourself leading this classroom intervention?” was measured 1 = *No*, 4 = *Maybe*, 7 = *Yes*.

**Motivation**. This was a five-item scale derived from questions used in Smith et al. (2007), and composed of the items “I think this is a valuable intervention,” “To me, this intervention is very interesting,” “To me, this intervention seems boring,” (reverse scored) and “I would enjoy using this intervention in my classes.” Each of these was measured on a scale from 1 = *Strongly disagree*, to 4 = *Neither agree nor disagree*, to 7 = *Strongly agree*. An additional item generated by the research team that that loaded onto this factor was, “What is your opinion of the UVI?” (1 = *Dislike a great deal*, 7 = *Like a great deal*).

**Expectancy-Value Mediators**

**Expectancies**. This was a three-item scale that measured perceived competence for implementing the UVI, adapted from Smith and colleagues (2007). Often used synonymously with expectancies for success, this measure was composed of the items, “Overall, I perceive the UVI is difficult to implement” (reverse scored), “The UVI seems easy to implement”, and “I would do a good job implementing the UVI in my classroom.” All items were measured on a scale from 1 = *Strongly disagree*, to 4 = *Neither agree nor disagree*, to 7 = *Strongly agree*.

**Value**. This was a six-item scale representing faculty perceptions of both the utility and attainment values of implementing the UVI, adapted from Hulleman and colleagues (2008). Whereas utility value refers to how a task can help achieve one’s long term goals, attainment value refers to a task’s power to reinforce one’s desired sense of identity. Reinforcing the theoretical notion that one’s identity (attainment value) can be strongly related to their most valued goals (utility value) (Wigfield & Eccles 1992), factor analysis suggested that the three items measuring each loaded together onto a single factor. The utility value items included “Implementing this classroom intervention will be useful for me in my career,” “Including this classroom intervention is useful for my students,” and “In general, using this intervention would not be useful to my students” (reverse-coded). Attainment value items included “Using an intervention to help students perform better in my courses is important to me,” “Increasing student interest in Biology is important to me,” and “Serving the needs of students is personally meaningful to me”. (1 = *Strongly disagree*, to 4 = *Neither agree nor disagree*, to 7 = *Strongly agree*.

**Cost**. This was a four-item scale of task effort cost adapted from Flake et al. (2015). Although Flake and colleagues have delineated multiple different types of cost, including loss of valued alternatives, emotional, and outside effort costs, the research team’s familiarity with biology faculty’s resistance to the UVI suggested task effort cost (e.g., time needed for grading) was the most relevant form of cost for the present study. Although we measured these other three cost measures, we chose to omit them from our model due to both the study’s goal of producing a parsimonious model and the statistical concerns of multicollinearity created when highly correlated predictor (i.e., multiple types of cost) are used to predict outcome measures. The final task effort cost scale was composed of the items “When I think about the hard work needed to implement the UVI, I am not sure that it will be worth it in the end,” “For me, adding these assignments just might not be worth the effort,” “Doing the UVI sounds like it really requires too much effort,” and “The UVI takes up too much time.” It was again measured with the scale 1 = *Strongly disagree*, to 4 = *Neither agree nor disagree*, to 7 = *Strongly agree*.

**Faculty Characteristics**

Several different types of faculty characteristics were collected. Personal and department variables were self-reported during the survey, whereas institutional characteristics were collected from the Integrated Postsecondary Education Systems (IPEDS) database.

***Personal Characteristics***

**Gender identity.** “What is your gender identity?” was self-reported in an open-entry field and initially recoded for “male,” “female,” or “non-binary.” This variable was ultimately treated as a binary measure (0 = *Male*, 1 = *Female*) because only 1 participant identified as non-binary, resulting in cell sizes too small for inclusion in analyses.

**Years teaching.** “How many years have you taught Biology?” was a self-reported in an open-entry field. Responses were coded onto a numeric scale.

**Racial/ethnic identity.** Faculty’s own racial/ethnic identity was self-reported in the survey with options available to check including: American Indian/Alaska Native, Black/ African American, Asian/ Asian American, Pacific Islander/ Hawaiian, Hispanic/Latino, Middle Eastern, White. This was recoded so that 0 = *Non-minoritized*, and 1 = *Minoritized* (American Indian/Alaska Native, Black/ African American, Hispanic/Latino, or Pacific Islander/Hawaiian), aligning with recent work suggesting these racial/ethnic identities are particularly underrepresented in faculty positions related to biological sciences (Valantine and Collins 2015).

***Departmental Characteristics***

**Size of class**. All Biology class sizes taught were self-reported in the survey, with options available to check including: Less than 50 students, 50-149 students, 150-299 students, 300+ students, and 500+ students. From this, the variable for largest class sized taught was created and faculty were categorized by the largest class size they indicated teaching. This was treated as a continuous variable in analyses.

**Percent workload dedicated to teaching**. This was self-reported in the survey as the percent of faculty’s working time that they spend on teaching. Faculty also had to enter percentages for time spent on research activities, and service activities, with the resulting sum required to equal 100. Because of this, percent workload dedicated to teaching is highly correlated with percent workload dedicated to research (*r* = -.79).

***Institutional Characteristics***

**Percent minoritized enrollment**. This was the percentage of undergraduate students from underrepresented backgrounds (Native American, Black, Hispanic/Latino) at the faculty’s institution (in 2018). This was too highly correlated with MSI status for both to be included in the model. The study’s results did not change depending on which variable was used.

**Percent of budget from research expenditures**. This was the percentage of the institution’s core budget devoted to research activities (in 2018).

# Section D. Analyses and Robustness Checks

**Exclusion criteria**

As stated in pre-registrations for our studies, faculty who volunteered to participate in our studies were required to pass eligibility, consent, awareness, and completion checks in order to be included in our study’s final analyses. Faculty participants who had not taught a biology class within the last three years or indicated they would not teach it soon were informed they were not eligible to complete the study, and those who indicated they did not consent at the beginning of the study were not asked further questions. Thereafter, participants were asked two pairs of awareness checks, the first to ensure they had understood the description of the utility value intervention (UVI) (i.e., “What is the main purpose of the UVI?”), and the second to ensure they were paying attention as they completed the remaining 20 minutes of survey questions (i.e., “Answer ‘completely true’ if you are reading”). Participants who incorrectly answered both questions in either pair were excluded. Finally, faculty who completed less than 60% of the study were excluded from analyses. These exclusion criteria were pre-registered at AsPredicted.org (see Section E for links to pre-registration details).

**Structural Equation Modeling**

Structural equation modeling was conducted using STATA 15. Full estimates corresponding to Figure 1 are presented in Table S3. In our final models, all faculty characteristic variables (exogenous variables) were correlated. In addition, the residual variances of all primary mediating variables (i.e., expectancies, value, cost) were correlated. Models were estimated using full information maximum likelihood. Reporting SRMR as a fit statistic was not supported by this estimation method. We therefore re-ran each of our models in this study using maximum likelihood estimation. The listwise deletion process resulted in the omission of 5% of our participants, acceptable SRMRs for all models, and substantially similar estimates throughout the model.

| **Table S3** | | | | | | | |
| --- | --- | --- | --- | --- | --- | --- | --- |
| Model 1 Results - Standardized Estimates for Expectancy-Value-Cost Model (Study 1) | | | | | | | |
| N = 422 | | | | | | | |
|  |  | Beta | SE | z | *p*-value | 95% Confidence interval | |
| *Predicting perceived competence* | |  |  |  |  |  |  |
|  | gender is female | 0.06 | 0.05 | -1.22 | 0.222 | -0.16 | 0.04 |
|  | faculty is minoritized | 0.11 | 0.05 | 2.23 | 0.026 | 0.01 | 0.21 |
|  | teaching experience | -0.10 | 0.05 | -2.14 | 0.033 | -0.20 | -0.01 |
|  | size of class | -0.27 | 0.05 | -5.06 | 0.000 | -0.38 | -0.17 |
|  | % teaching focus of faculty | 0.00 | 0.05 | 0.08 | 0.938 | -0.10 | 0.10 |
|  | % minoritized of undergrad | 0.02 | 0.05 | 0.32 | 0.748 | -0.08 | 0.11 |
|  | % of budget to research | -0.05 | 0.06 | -0.81 | 0.418 | -0.16 | 0.07 |
|  | intercept | 4.41 | 0.26 | 16.71 | 0.000 | 3.90 | 4.93 |
|  |  |  |  |  |  |  |  |
| *Predicting utility/att. value* | |  |  |  |  |  |  |
|  | gender is female | 0.15 | 0.05 | -2.89 | 0.004 | -0.24 | -0.05 |
|  | faculty is minoritized | 0.06 | 0.05 | 1.28 | 0.200 | -0.03 | 0.16 |
|  | teaching experience | -0.05 | 0.05 | -0.99 | 0.323 | -0.15 | 0.05 |
|  | size of class | 0.05 | 0.06 | 0.93 | 0.352 | -0.06 | 0.16 |
|  | % teaching focus of faculty | 0.03 | 0.05 | 0.60 | 0.552 | -0.07 | 0.13 |
|  | % minoritized of undergrad | 0.06 | 0.05 | 1.23 | 0.218 | -0.04 | 0.16 |
|  | % of budget to research | -0.13 | 0.06 | -2.13 | 0.033 | -0.24 | -0.01 |
|  | intercept | 9.66 | 0.40 | 23.87 | 0.000 | 8.87 | 10.46 |
|  |  |  |  |  |  |  |  |
| *Predicting task effort cost* | |  |  |  |  |  |  |
|  | gender is female | -0.09 | 0.05 | 1.69 | 0.092 | -0.01 | 0.18 |
|  | faculty is minoritized | -0.05 | 0.05 | -1.07 | 0.284 | -0.15 | 0.05 |
|  | teaching experience | 0.10 | 0.05 | 1.93 | 0.053 | 0.00 | 0.19 |
|  | size of class | 0.14 | 0.06 | 2.42 | 0.016 | 0.03 | 0.25 |
|  | % teaching focus of faculty | 0.05 | 0.05 | 0.99 | 0.322 | -0.05 | 0.15 |
|  | % minoritized of undergrad | -0.07 | 0.05 | -1.35 | 0.177 | -0.17 | 0.03 |
|  | % of budget to research | 0.02 | 0.06 | 0.25 | 0.802 | -0.10 | 0.13 |
|  | intercept | 1.82 | 0.28 | 6.58 | 0.000 | 1.28 | 2.36 |
|  |  |  |  |  |  |  |  |
| *Predicting intrinsic interest* | |  |  |  |  |  |  |
|  | perceived competence | 0.14 | 0.05 | 2.99 | 0.003 | 0.05 | 0.24 |
|  | utility/att. Value | 0.56 | 0.03 | 16.40 | 0.000 | 0.49 | 0.62 |
|  | task effort cost | -0.12 | 0.05 | -2.39 | 0.017 | -0.22 | -0.02 |
|  | intercept | 0.94 | 0.48 | 1.94 | 0.053 | -0.01 | 1.88 |
|  |  |  |  |  |  |  |  |
| *Predicting implementation intentions* | | |  |  |  |  |  |
|  | perceived competence | 0.17 | 0.05 | 3.44 | 0.001 | 0.07 | 0.26 |
|  | utility/att. Value | 0.05 | 0.05 | 0.94 | 0.346 | -0.05 | 0.14 |
|  | task effort cost | -0.22 | 0.05 | -4.34 | 0.000 | -0.31 | -0.12 |
|  | intrinsic interest | 0.43 | 0.05 | 9.19 | 0.000 | 0.34 | 0.52 |
|  | intercept | 0.53 | 0.47 | 1.12 | 0.262 | -0.40 | 1.45 |
| Note. Residual variances of all mediators (Expectancies, Value, Costs) are correlated. SRMR is not calculated because model uses full information maximum likelihood estimator. When using maximum likelihood (listwise deletion), sample size is reduced by 19 cases (5%), estimates do not significantly change, and SRMR = 0.017. | | | | | | | |

The moderate negative correlation between expectancies and cost (*r* = -.68) led to concern about multicollinearity when predicting intrinsic interest and implementation intentions. We therefore also tested a model that removed expectancies and a model that removed cost (see Figure S3 and S4). As anticipated, the omission of either expectancies or cost made the coefficient magnitude of the other greater when predicting our outcomes. However, because this was essentially all that changed (model fit and direction of effects did not significantly change), we focus on the direct and indirect effects of the full expectancy-value-cost model.

**Figure S3.** Re-running Model 1 after removing cost from the model. Insignificant paths not shown. Exogenous variables are all correlated. Residual variances of all mediators (Expectancies, Value) are correlated. SRMR is not calculated because model uses full information maximum likelihood estimator. When using maximum likelihood (listwise deletion), sample size is reduced by 19 cases (5%), estimates and fit statistics do not significantly change, and SRMR = .018.

**Figure S4.** Re-running Model 1 after removing expectancies from the model. Insignificant paths not shown. Exogenous variables are all correlated. Residual variances of all mediators (Expectancies, Value) are correlated. SRMR is not calculated because model uses full information maximum likelihood estimator. When using maximum likelihood (listwise deletion), sample size is reduced by 19 cases (5%), estimates and fit statistics do not significantly change, and SRMR = .024.

**Mean Differences**

After data suggested five faculty characteristics were negatively associated with implementation intentions, we conducted t-tests to confirm that mean differences between these groups were statistically significant. Women-identified (*n* = 211), men-identified (*n* = 1199), minoritized (*n* = 44) and non- minoritized (*n* = 362) were all easily categorized. For the remaining variables, which were continuous, grouping participants required establishing cutoffs at one standard deviation below and one standard deviation above the mean (means and standard deviations for each measure available in Table 1 of the manuscript). Therefore, few years teaching (*n* = 59) were those with less than 5.7 years teaching experience, whereas many years teaching (*n* = 62) were those with at least 25.0 years teaching experience. Small class (*n* = 169) were those teaching classes of 50 or fewer, whereas large class (*n* = 104) were those teaching classes of 150 or more. Low research (*n* = 152) were those at universities that reported 0% of their budgets to research expenditures, whereas high research (*n* = 81) were those at universities that reported at least 13.2% of their budgets to research expenditures. We note that all 81 faculty from “high research” universities were at doctoral-granting institutions recognized as “very high research activity” (R1) or “high research activity” (R2) schools, whereas 76% of faculty from “low research” universities were at Baccalaureate or Master’s institutions. T-tests showed that, except for faculty from a minoritized background, all of these differences were statistically significant at the *p* < .01 level.

**Figure S5.** Means and standard errors of implementation intentions by faculty, departmental, and institutional characteristics. Minoritized/non-minoritized is the only group difference not statistically significant at the *p* < .01 level.

# Section E. Larger Study Details

The participants in this study spanned several waves of data collection designed to test subtle manipulations of messaging describing the UVI. These were intended to alter perceptions of the UVI (i.e., motivation, cost, implementation intentions). The only differences in the materials introduced by the experiments were whether subtle messages manipulated:

**Experiment 1 – Who the UVI helps.** Helps struggling students, Helps diverse students, no information (Control).

**Experiment 2 – Who endorses the UVI.** Faculty endorse, funding agencies endorse (like NSF and NIH), no information (Control).

**Experiment 3 – Who feels social pressure to adopt the UVI.** Universities have a responsibility, scientists have a responsibility, no information (Control)

Because participants were randomly assigned to conditions, potential moderators of how the UVI’s presentation affected faculty’s subsequent motivation to try it (e.g., gender identity, years teaching, ethnic identity) were uncorrelated with condition assignments. Pre-registered analyses showed that such subtle manipulations did not significantly alter faculty’s expectancies, value, cost, motivation, or implementation intentions regarding the UVI. In line with our pre-registrations, we tested these using ANCOVAs with Bonferroni adjustments were used for inputs 1 (<https://aspredicted.org/blind.php?x=au4qy6>) and 2 (<https://aspredicted.org/blind.php?x=ep9d4z>), and multiple regressions with planned contrasts for input 3 (<https://aspredicted.org/blind.php?x=yi9v5p>). After conducting a covariate analysis, all models controlled for gender identity, years teaching (continuous variable), and largest class size (measured in the same fashion as described in the Measures section of this manuscript). Exemplar graphs below are meant to demonstrate that several outcome measures in the present paper (i.e., cost, motivation, implementation intentions) did not significantly differ based on these experimental conditions in any of the three experiments.

Experiment 1 Experiment 2 Experiment 3

**Figure S6.** Results of preregistered experimental analyses. Results show non-significant effects of manipulating presentation of the UVI on three exemplar outcomes: cost, motivation, and implementation intentions.

# Section F. Additional Descriptive Statistics

| **Table S4** | | | | | | |
| --- | --- | --- | --- | --- | --- | --- |
| Associations of Gender Identity with Other Faculty Characteristics | | | | | | |
|  |  | Mean | SE | SD | t | p |
| Years teaching | |  |  |  |  |  |
|  | women-identified | 12.82 | 0.56 | 8.10 |  |  |
|  | men-identified | 17.65 | 0.72 | 10.18 |  |  |
|  | difference | -4.83 | 0.91 |  | -5.33 | <.001 |
|  |  |  |  |  |  |  |
| Size of class | |  |  |  |  |  |
|  | women-identified | 1.93 | 0.07 | 1.01 |  |  |
|  | men-identified | 1.96 | 0.07 | 0.98 |  |  |
|  | difference | -0.03 | 0.10 |  | -0.32 | 0.751 |
|  |  |  |  |  |  |  |
| % Workload dedicated to teaching | | | |  |  |  |
|  | women-identified | 66.62 | 1.48 | 21.46 |  |  |
|  | men-identified | 59.11 | 1.58 | 22.23 |  |  |
|  | difference | 7.51 | 2.16 |  | 3.48 | <.001 |
|  |  |  |  |  |  |  |
| % minoritized enrollment | |  |  |  |  |  |
|  | women-identified | 27.49 | 1.33 | 19.34 |  |  |
|  | men-identified | 28.30 | 1.30 | 18.30 |  |  |
|  | difference | -0.81 | 1.86 |  | -0.43 | 0.666 |
|  |  |  |  |  |  |  |
| % of budget from research expenditures | | | |  |  |  |
|  | women-identified | 5.11 | 0.50 | 7.31 |  |  |
|  | men-identified | 5.60 | 0.54 | 7.60 |  |  |
|  | difference | -0.49 | 0.74 |  | -0.66 | 0.510 |
| *Note*. N = 211 for women-identified and n = 199 for men-identified | | | | | | |

| **Table S5** | | | | | | |
| --- | --- | --- | --- | --- | --- | --- |
| Associations of Racially Minoritized Status with Other Faculty Characteristics | | | | | | |
|  |  | Mean | SE | SD | t | p |
| Years teaching | |  |  |  |  |  |
|  | non-minoritized | 15.44 | 0.51 | 9.64 |  |  |
|  | minoritized | 13.00 | 1.32 | 8.74 |  |  |
|  | difference | 2.44 | 1.52 |  | 1.60 | 0.110 |
|  |  |  |  |  |  |  |
| Size of class | |  |  |  |  |  |
|  | non-minoritized | 1.91 | 0.05 | 0.97 |  |  |
|  | minoritized | 2.34 | 0.17 | 1.16 |  |  |
|  | difference | -0.43 | 0.16 |  | -2.71 | 0.007 |
|  |  |  |  |  |  |  |
| % Workload dedicated to teaching | | | |  |  |  |
|  | non-minoritized | 63.57 | 1.15 | 21.79 |  |  |
|  | minoritized | 57.50 | 3.58 | 23.76 |  |  |
|  | difference |  | 6.07 | 3.51 | 1.73 | 0.085 |
|  |  |  |  |  |  |  |
| % minoritized enrollment | |  |  |  |  |  |
|  | non-minoritized | 26.17 | 0.90 | 17.08 |  |  |
|  | minoritized | 41.14 | 3.85 | 25.56 |  |  |
|  | difference | -14.96 | 2.90 |  | -5.15 | <.001 |
|  |  |  |  |  |  |  |
| % of budget from research expenditures | | | |  |  |  |
|  | non-minoritized | 5.01 | 0.38 | 7.15 |  |  |
|  | minoritized | 5.38 | 0.37 | 7.47 |  |  |
|  | difference | -3.42 | 1.18 |  | -2.90 | 0.004 |
| *Note*. N = 361 for non-minoritized and n = 44 for minoritized | | | | | | |

**References**

National Science Foundation (2019). Women, minorities, and persons with disabilities in science and engineering: Table 9-29. Available at <https://ncses.nsf.gov/pubs/nsf21321/report/academic-careers#representation>. Accessed March 24, 2022.
